# Supplementary material for: Paranoia, self-deception and overconfidence
Source: PLoS Comput Biol. 2021 Oct 7;17(10):e1009453. doi: 10.1371/journal.pcbi.1009453 (PMC8525769; doi:10.1371/journal.pcbi.1009453)
Supplement: S1 Table — (PDF) [file pcbi.1009453.s001.pdf]

S1 Table

	Low paranoia (n=469)	High paranoia (n=194)	Statistic	P-value
**Age**	38.6 (10.96)	35.92 (9.28)	-3.1899(396.16)	0.001537
**Gender**			5.49(3)	0.1393
% Female	42.86%	47.42%		
% Male	54.16%	51.03%		
% Other/not specified	2.98%	1.52%		
**Education**			53.39(7)	3.1e-9
% High school degree or equivalent	9.38%	7.22%		
% Associate degree	12.16%	6.7%		
% Bachelor's degree	43.28%	44.85%		
% Master's degree	7.68%	26.2%		
% Doctorate or professional degree	1.7%	1.03%		
% Completed some college	22.43%	13.85 %		
% Other/not specified	3.41%	0%		
**Ethnicity**			21.965(2)	1.7e-5
% Hispanic or Latino	8.1%	20.6%		
% Not Hispanic or Latino	89.13%	78.35%		
% Not specified	2.77%	1.03%		
**Race**			36.19(5)	8.69e-7
% White	76.76%	65.98%		
% Black or African American	9.38%	22.16%		
% Asian	4.26%	5.67%		
% American Indian or Alaska Native	0.21%	2.57%		
% Multiracial	6.18%	3.61%		
% Other/not specified	3.2%	0%		
**Income**			17.15(6)	0.0087
% Less than $20,000	18.55%	12.88%		
% $20,000 to $34,999	17.91%	17.01%		
% $35,000 to $49,999	16.41%	22.16%		
% $50,000 to $74,999	24.73%	30.93%		
% $75,000 to $99,999	11.94%	13.92%		
% Over $100,000	6.18%	2.58%		
% Decline to answer	4.3%	0.52%		
**Psychiatric Diagnosis**			53.39(7)	3.1e-9
% No psychiatric diagnosis	61.19%	36.08%		

% Schizophrenia spectrum	0.43%	0.515%		
% Anxiety/Depression only	8.32%	13.92%		
% Bipolar disorder	1.49%	0%		
% Multiple/not specified	10%	9.28%		
% Personality disorder	0.64%	3.6%		
% Decline to state	17.9%	36.6%		
**% Medicated**	6.39%	5.15%	0.186(1)	0.6659
**BAI**	5.77(7.83)	21.68(15.49)	13.6(234.84)	<2.2e-16
**BDI**	8.08(10.49)	23.38(15.39)	12.68(270.18)	<2.2e-16
**GPTS**	0.22(0.4)	2.26(0.69)	38.55(252.11)	<2.2e-16
