# Supplementary material for: Paranoia, self-deception and overconfidence
Source: PLoS Comput Biol. 2021 Oct 7;17(10):e1009453. doi: 10.1371/journal.pcbi.1009453 (PMC8525769; doi:10.1371/journal.pcbi.1009453)
Supplement: Attachment — Submitted filename: reviewer-comments-response-8-23.docx [file pcbi.1009453.s012.docx]

Reviewer's Responses to Questions

**Comments to the Authors:**

Reviewer #1: This is an excellent and timely paper. The authors set out to investigate the interaction of social context (cooperation vs competition) with perceptual belief updating and trait paranoia. To do so they designed an elegant joint perception experiment in which participants view visual morphs and make bets as to which morph is dominate, either competing through deception or cooperating through self-deception. The authors then apply this task in a large online cohort, and model responses using an adapted version of the hierarchical gaussian filter, operationalizing the decision process as a dual stream processes in which social and perceptual beliefs are integrated together. This approach reveals interesting differences in choice behavior and underlying computational mechanisms, which are further replicated and extended in a second follow up experiment manipulating choice accuracy. Overall the findings are robust and interesting, the modelling appropriate, and the manuscript extremely well written. I have little to add to this manuscript other than a curiosity about the confidence ratings, which here are largely used to titrate behavioral responses. However, adding metacognitive modelling at this stage would probably bloat an otherwise extremely tight paper, so this is just my curiosity. I have only one major comment for consideration:

1. If I understand the model validation steps correctly, the authors do an excellent job of motivating a complex family of models determining the complexity of the winning dual stream model. They also compare this model to a non-bayesian alternative using model comparison, and then conduct a posterior predictive check by correlating simulated data generated by this model to empirically fitted data. My only question is if this final step is sufficient - from the supplementary figure there appears to be some **potential deviance between the two parameter set**. Is there a way to quantify this directly? I could imagine for example using a cross-validation procedure, e.g. fitting the models to a subset of the data and then predicting "hold out" data to determine empirical out of sample prediction accuracy. However, this may be unnescessary if I have misunderstood some aspect of the model validation procedure.

**First, thank you for the encouraging comments.**

**On the need for outsample prediction; since our results are focused on group differences, which were maintained with the simulated data, we do not think direct quantification is necessary or will be particularly informative. Other published work with HGF models tends to employ simulated responses and parameter recovery via those simulated responses (Henco et al., 2020; Diaconescu et al., 2014). We followed Palminteri et al’s (2017) suggestions and sought quantitative and qualitative reassurance that we have the right model and we are not overfitting. We hope the reviewer agrees.**

**Reviewer #2:** 

The authors describe data from a study in which they examined perceptual inference in participants under conditions in which they did or did not get advice from a confederate who was either collaborating or competing with them. Data was analyzed at both the direct behavioral level and with an HGF.

I think this study has a lot going for it. The basic task design and the results (as far as I can sort them out) are quite interesting. In general this is also an interesting question of relevance to psychopathology. However, many things are not clearly explained. I had a hard time understanding the task design, the behavioral variables being analyzed and, more fundamentally, the hypotheses and how the overall approach was addressing those. Although I could understand enough to generally follow the thread of the results, I think a much more detailed account of the task, the behavioral data analyzed, and a more clear account of the findings would substantially improve this manuscript.


Comments
1. The details of the task were unclear, as was the definition of self-deception. For some of the ANOVA models it wasn’t clear what the dependent variable was. For example, “Analysis of variance revealed a main effect of paranoia (high or low), a main effect of group (competition or collaboration) but no paranoia by group interaction for self-deception.” I don’t know what is being analyzed. I think the behavioral data should be shown more clearly. And then the metrics that are derived from that data that serve as dependent variables should be explained clearly and illustrated. Best to do this in the results, but the methods could also use more detail on these points.

**We regret that our task was unclear. In the revised manuscript, we have strived for clarity – particularly around the definition of self-deception. The ANOVA tested whether self-deception scores were dependent on paranoia (high or low) and group (competition or collaboration). We have added all the dependent variables to the descriptions of the ANOVAs and ANCOVAs in the behavioral section (lines 287-307). Some additional details have been added to the methods section about the task (Lines 128-142) (the task was originally published in Leong et al., 2019). We have added a table defining self-deception in terms of patterns of behavioral responding, and we outline where the behavioral metrics are derived from in Figure 1 (Panel E). We have added additional description of the behavioral metrics in the results section.**

2. It would be useful to show and reference a figure for this when it is stated, “the probability of responding scene followed an s-shaped psychometric curve, indicating that in general, participants were able to categorize the chimeras accurately.”

**We have added the psychometric plots in Figure 1; it is now in panel C. As the reviewer will see, participants were generally accurate.**

3. For this section, “The difference between groups remained significant when we examined confidence-normalized self-deception score (F(1, 620) = 58.0612, pbonf= 2.8659e-13,   2 184 = 0.0859; Figure 3B, C). We also found that the cooperation group had increased confidence-weighted self-deception (F(1, 620) = 15.0085, pbonf=3.442e-4,   2 186 = 0.02673) – people were more likely to confidently self-deceive to conform to their partners’ bet in the cooperation group relative to defecting from the bet in the competition group. The absence of group by paranoia interaction, suggests that centering in vs out-group membership was not differentially impacted by paranoia (Figure 3D-E).” What is confidence-normalized self-deception score? What is “centering in vs out-group…”?

**We have rewritten this section (now on lines 288–309). The confidence-normalized self-deception is the raw self-deception score (the percentage of responses that were self-deceptive, defined by the response pattern in Figure 1E) multiplied by the mean confidence on self-deceptive trials normalized by the participants mean confidence during the initial classification phase (C1). This metric captures the highest self-deception when the individual self-deceives with increased confidence over their individual confidence baseline. We have also referenced the equation in the methods that explains confidence-normalized self-deception. With regards to statement “centering in vs out-group membership was not differentially impacted by paranoia” – we had meant that accounts of paranoia which focus on coalitions and group identity were not supported by our data. We have modified the language in that section to clarify the predictions of those models and how those predictions were not bourne out by our data.**

4. The model should also be explained in the results. Some comments on what the variables measure. I know some of this is in the methods, but it would be much easier to give some background on the model and the variables in the results. The technical definitions can be given in the methods. It’s best to present the results and then summarize what they mean in each paragraph. It is also helpful if you can go back to the raw data and compute metrics directly on the raw data that show the effects extracted by the model. Also, I assume the model is mainly for studying learning, but the learning effects were buried and so it was not clear how participants updated belief estimate over trials on the basis of past feedback. It is also not clear why there was a comparison to Rescorla-Wagner, since such a model has no way of incorporating ambiguity in the cues, and the RW model is also no really applicable since the cues contain the information about the relevant behavior, whereas RW is more learning associations between values and arbitrary cues. This is not really a meaningful comparison.

**We believe that the explanation of the model is better left in the methods as it is very technical in nature. We have added a paragraph in the results explaining our reasoning behind using this specific type of modeling (lines 361–321).**

**The descriptions about the meaning/interpretation of the relevant model parameters has been updated in the results section. We have also added Supplementary table 3, which lists the model parameters and their descriptions.**

**The equations for belief updating are in the Methods section (Equations 2-5, 7,8). We have added the aforementioned section in the results about why we use a learning model on a task that is not a learning task.**

**The RW model is used to show how a fully non-normative model is unable to capture the behavioral effects of interest. The arbitrary cue in this case is the bet. While the bet contains no true informational value, both our behavioral and modeling results indicate that participants were influenced by it. Thus, we assessed if a RW model would explain the associations between the choices and the bets.**

“It is also helpful if you can go back to the raw data and compute metrics directly on the raw data that show the effects extracted by the model.”

**We achieve this in our model validation results. The model produces self deception and the simulated data recapitulate the critical group differences.**

5. The introduction could use a rewrite. It does not flow well. But more importantly, it does not setup the specific experiment that was done, or why it was done. What are the hypotheses and how does the experiment address these?

**We have added some additional details in the introduction about our specific hypotheses. In brief, being aware of perceptual, learning, and coalitional accounts of paranoia, we sought to adjudicate using a task that engaged coalitional cognition, and a modeling approach that pitted social and non-social processes against one another to see which better accounted for paranoia.**

**Reviewer #3:**

This is an interesting study of how perceptual beliefs can be swayed by the view of a confederate, in subjects with low or high paranoia levels. The authors used an online task in which a chimeric image is shown and a participant must rate it as majority face or house. A confederate then 'bets' on the outcome in a second condition, and the rating is made again, with a bonus for both being correct in the collaborator condition, or for correctly identifying they are incorrect in the competitor condition. A Bayesian model was used to evaluate the relative contributions of previous trials and social information to decisions made.

The authors found:
- Participants were more likely to agree with collaborators' bets and disagree with opponents' bets
- This was especially the case for high paranoia participants (but not when bets were more accurate), but no interaction with competition/collaboration was found
- The modelling found the cooperation group had stronger initial faith in the bet
lower recency bias (bigger omega_ns) about the image in high paranoia (replicated in another sample with higher bet accuracy)
>updating about the accuracy of the bet (bigger omega_s) in high paranoia
- Lower recency bias about the image correlated with 'self-deception' - this was stronger in high paranoia
- Lower recency bias appeared to relate to paranoia rather than anxiety, although these were correlated

Overall the paper uses a clever task and sophisticated modelling to show that paranoia is associated with less trial-to-trial influence of the image, and more influence of the confederate (in terms of updating about bet accuracy). I do have some concerns about the results, however: the main ones are i) whether this pattern of results could also be explained by inattentive responding? ii) I find some of the statements in the paper rather strong given the evidence - especially the term 'self-deception', and some assertions about paranoia. These are detailed below:

p3 - Were the subjects told that the reward-maximising strategy is to judge accurately?

**No, they were not instructed explicitly. They were informed that their goal was to correctly classify as many images as possible. We have included this information explicitly in the methods section now (lines 134-135).**

Also, describing a response that changes according to the opponent/collaborator's bet as "self-deceptive" doesn't make sense to me. If I adjust my opinions having heard someone else's views, am I deceiving myself? This seems too strong and loaded a term to apply to this effect. For example, on p7, the finding that "self-deception... was driven by perceived unreliability of ones' own choices" could better be described as "loss of confidence in oneself increases the influence of others".

**We are using the framework from Mijovic-Prelec and Prelec (2010) to define self-deception (the table for responding patterns is now included in Figure 1). It is not just about hearing other’s views – it is about utilizing irrelevant information to make a decision (that rationally should be discarded). We feel it appropriate to suggest that self-deception occurs when one adjusts their opinions to incorporate useless information when it is against their economic interest to do so. If participants relied only on the informational value of the opponent or collaborator, they would be equally likely to respond in line with the collaborator as the opponent. We see a bias (where they are more likely to agree with the collaborator and disagree with the opponent) which tells us that this is more than just adjusting your viewpoint with new information. Self-deception can be conceptualized as a form of motivational bias.**

p4 - The description of the model is quite confusing and scanty. It needs to be much clearer. What is zeta? What is eta? What is w in Figure 2A? Is one of these the recency bias, as that term doesn't appear in Supp Table 3? Omega_ns doesn't appear in any equation that I found - yet it is the parameter behind the key group effect?

**We have added Supplementary Table 3 that provides the definition of all the parameters. The “w” in Figure 2A is an intermediate variable (not a parameter) that is computed to combine the 2 streams of information. We have removed it from the model schematic in Figure 2.**

**The recency bias is not a parameter. It is an equation (Eq. 6), that is now included in the methods section explicitly on lines 217­–221. We have added the equation for omega-ns directly to the methods section (since it is in the backend of the HGF) (Equation 2).**

p7 - Given there was no trial-to-trial dependency in this task, it seems unwarranted to say that a lower recency bias in paranoid individuals might reflect a "lack of trust in one's abilities", given that having no recency bias at all is actually optimal here, if I understand correctly?

**The recency bias was not lower in paranoid individuals ­– the recency bias is not a parameter but is a value that changes for every trial. We have added the equation for the recency bias in the methods to address this. We have updated the interpretation about omega-ns in the results section (lines 344–353) to clear up the relationship between this parameter and the recency bias (which is also illustrated in Figure 2C).**

**We found in the high paranoia group that they had an elevated omega-ns. Having a greater omega-ns causes the recency bias for that trial to impact the classification less (and the higher-level associations about the images will impact the classification more).**

**In summary, the actual recency bias is not altered – the contribution of the recency bias to the choice is decreased in high paranoia participants relative to the contribution of the 2^nd^ level belief. This effect is maximized when the recency bias is 0, which occurs when the image in the previous trial is very ambiguous.**

Also, a persuasive recent preprint by Zorowitz et al ([psyarxiv.com/rynhk](http://psyarxiv.com/rynhk)) showed that inattentive responding can induce spurious results in online studies. Can the authors reassure the reader that this could not explain their results? It seems to me that more inattentive players are likely to show less influence of recent trials, more influence of a confederate, and are more likely to score highly on anxiety/paranoia scales. The fact that no interaction with competition/collaboration is a bit concerning here - an interaction would make the results more specific to paranoia itself. Are any of these effects highly unlikely to be accounted for by inattentive responding?

**We appreciate your insight here about this potential issue. We cannot directly use the suggestions in the preprint, as they would involve adding inattention items to our scales. We now acknowledge this as a limitation of our work. However, we do not think our specific effects could be accounted for by inattentive responding. Inattentive responding should fall randomly across scales, and would not yield the specific associations between self-rated experiences and beliefs, and participant behavior. Furthermore, if our high paranoia participants are merely inattentive, we might expect those in the competition condition to simply agree with the confederate more. We see the opposite effect.**

**The authors of the preprint specifically note that in their sample, they found that the rate of participants who had clinically elevated levels of depression and anxiety was much higher than the national average, and these levels dropped after removing participants for inattentive responding. In contrast, our data showed levels of anxiety and depression far beneath the national average, again suggesting that our participants were answering in earnest and our results are not as inflated as those reported in the pre-print.**

p8 - What exactly would the 'coalitional cognition' account of paranoia predict, and why?

**The coalitional cognition account would predict that there would be an interaction effect between group and paranoia. For example, we would expect to see increased self-deception in the competition group in high paranoia participants relative to the high paranoia cooperation group. We did not find any behavioral or parameter interaction effects between paranoia and experimental condition.**

p9 - Why is it not the case that high paranoia individuals just have a more uncertain model? Hence self-confidence is lower and ability is diminished, but others' influence over them is stronger? What exactly justifies the added interpretation that overconfidence and 'self-deception' are *protecting* against negative self-image? Rather than just inevitable consequences of having a noisier model?

**We don’t necessary think that a more uncertain model would lead to an increased influence of others over them. Uncertainty might drive them to resist help/assistance from others, if they prefer to make their own decisions (for example). Furthermore, a merely noisy model would not necessarily engender higher confidence in self-deceptive choices – if low self-confidence is being driven by an uncertain model, we would not expect to see increased reported confidence on self-deceptive trials (we would expect to see random fluctuations in reported confidence). This overconfidence (above their own individual baseline) combined with the increased utilization of the perceived tendencies (omega-ns) in classification is protective in that it allows them to ignore their low self-confidence (by reporting the opposite) while disregarding the sensory evidence (thus protecting themselves from recognizing their low ability).**

p10 - It is odd to perform a multiple regression showing that both paranoia and anxiety significantly correlate with omega_ns, but not to report the actual results in terms of betas, confidence intervals etc. If the regression is abandoned because of multicollinearity issues, the variance inflation factors should be reported to justify this. What was the cut-off for 'high' vs 'low' anxiety and how was it chosen? Also, the authors state "paranoia appears more responsible for the group differences in self-deception and the associated model parameters" - but omega_ns (unless I misunderstand) concerns the recency bias, not self-deception: its relationship with self-deception is indirect. To make the statement above, this analysis should have looked at self-deception, paranoia and anxiety directly?

**We have added the beta and confidence values to the multiple regression. The regression was indeed abandoned due to multicollinearity, so we have performed a Farrar-Glauber test to justify this. The clinical cutoff values for the BAI scores have been added and cited. Omega-ns was not the recency bias, and hopefully we have cleared that up in the comments above and in adding the definition of recency bias to the methods. While doing a regression directly for self-deception, BAI score was not even a significant predictor, which is why this relationship was never explored in the behavioral results. Omega-ns is a latent factor that seemed worth exploring in relation to the other scales.**

p10 - the first process driving increased self-deception in high paranoia is described as "an underweighting of current sensory inputs relative to the prevailing tendencies from recent trials", but to me a reduced recency bias in that group ought to mean current inputs are *less* influenced by prevailing tendencies?

**The recency bias relates to more influence from the recent stimuli – it is not related to the prevailing tendencies, and the value of the variable for the prevailing tendencies is not used in the computation of the recency bias (omega-ns is not used in the computation of the recency bias, see equation 6 in the methods section). The “prevailing tendencies” are the tendency of choice rather than the tendency of the trial.**

p11 - is it not too much to assert that "we found... paranoia can indeed by explained in Bayesian terms" given that no interaction with condition was found? i.e. the effect of competition was not greater in these subjects? Surely an account of paranoia must explain the particular direction of the effect?

**The interaction between condition and paranoia group would support the evidence of the coalitional cognition account. We are arguing directly against that, and yes, we are saying that an account of paranoia can actually be domain general and thus we do not see a direction in that effect. We have softened the language here.**

Minor points

p2 - I cannot find the Hagen (2008) reference but does it really show that too much self-deception leads to delusional beliefs? Also the evidence that paranoia protects self-esteem is pretty weak, all told (Murphy et al, 2018, Lancet Psych), so the claims about paranoia causing "direct inflation of self-image" are not realistic.

**The chapter outlines a theoretical model which attempts to account for delusions (in the context of delusional disorder) in terms of the secondary gain that might arise from successfully deceiving those around you into believing your delusion too. In order to achieve this gain, Hagen argues, after Trivers and others, that one must deceive oneself to better deceive others.**

**Here is a link to the chapter:**

[**http://citeseerx.ist.psu.edu/viewdoc/download?doi=10.1.1.650.1559&rep=rep1&type=pdf**](http://citeseerx.ist.psu.edu/viewdoc/download?doi=10.1.1.650.1559&rep=rep1&type=pdf)

**We agree that the inflationary account of paranoia has not garnered extensive support. However, we show in our own data that paranoia may have its roots in concerns about ones’ own performance (relative to the advisors), and, in highly paranoid people those concerns seem to be related to the frequency of overconfident self-deceptive responses. Thus, we conclude that our findings are consistent with that framework and inconsistent with a coalitional account.**

Supp Fig 1 - the correlations are not reported? Also the recovered parameters end up squashed into a smaller range, although I suppose this doesn't matter so much if one is only interested in group differences. Also for mu0_2, this should be transformed and the correlation computed in transformed space as the vast majority of the datapoints are in the cloud just above zero.

**It appears to be standard practice in the parameter recovery to only report p-values, but we have added the r-values to the revised manuscript. We agree, as we are only interested in the group differences the range is okay. In addition, the HGF literature shows that can be common in parameter recovery, particularly for parameters of the upper level (see Henco 2020 for an example). We log transformed mu0_2 and have reported the correlation for the transformed data.**

**References**

**Diaconescu AO, Mathys C, Weber LA, Daunizeau J, Kasper L, Lomakina EI, et al. Inferring on the intentions of others by hierarchical Bayesian learning. PLoS Comput Biol. 2014;10(9):e1003810. doi: 10.1371/journal.pcbi.1003810.**

**Henco L, Diaconescu AO, Lahnakoski JM, Brandi ML, Hormann S, Hennings J, et al. Aberrant computational mechanisms of social learning and decision-making in schizophrenia and borderline personality disorder. PLoS Comput Biol. 2020;16(9):e1008162. doi: 10.1371/journal.pcbi.1008162.**

**Palminteri S, Wyart V, Koechlin E. The Importance of Falsification in Computational Cognitive Modeling. *Trends Cogn Sci*. 2017;21(6):425-433. doi:10.1016/j.tics.2017.03.011**
