# Supplementary material for: Paranoia, self-deception and overconfidence
Source: PLoS Comput Biol. 2021 Oct 7;17(10):e1009453. doi: 10.1371/journal.pcbi.1009453 (PMC8525769; doi:10.1371/journal.pcbi.1009453)
Supplement: Attachment — Submitted filename: reviewer-comments-9-8-updated.docx [file pcbi.1009453.s013.docx]

Reviewer #2: The authors have substantially improved the manuscript. The intro could still be written a bit more fluently, particularly the first paragraph. But it’s better. I am still not clear on a few details of the experiment.

Specifically, I assume that in C1, there is no partner response? The participant is making choices but they do not see a choice made by a partner? This should be made clear. I assume there was no partner, but then I did not understand this sentence, “In experiment 1, the bets were correctly exactly 50% of the time.” Is this referring to the partner’s bets in C2? Or what is this referring to?

**Yes, this is referring to the bets in C2. There are no bets in C1 which we have now explicitly clarified in the methods (page 3, 141-142).**

The payoff matrix should be stated in the methods, with a reference to Figure 1B. This is important. Also, in the 50% condition, it is not clear how the subject should respond. Specifically, I don’t think the following comment is clear for the 50% condition, “Crucially, the reward maximizing strategy is to classify the images correctly.” This would only be true if the partner really was guessing and therefore correct at chance level. So, the subject has to assume this. If the subject assumes the partner is above chance it would make sense to respond with or against the partner depending on the condition. It is possible then that paranoia changes the subject’s assessment of whether the partner is correct or not, and the subject is behaving optimally, as opposed to in a self-deceptive way.

**The reviewer raises an interesting point, however, the participants do not know that some of the images are exactly 50% face and 50% scene. They might perceive even a 50/50 chimera as more face or more scene. Crucially, they see the exact same images in C2 as they did in C1. Therefore, on the 50% condition, if they are aiming for accuracy (as we instructed them to, see line 137-139), they should respond during C2 (with partner) the same way as they did in C1 (in the absence of partner).**

**The “partner” is in fact guessing – their advice is 50% accurate. Since there is no reason, nor instruction, to believe that the partner has any more information than the participant, participants might detect that the partner is guessing and disregard the partner’s advice (whether competitor or collaborator). We have added the following sentence to the methods to make this very clear:**

***“Participants are not told that the partner or opponent has been given any more information than them, and importantly, the bet is made before the image for the trial is shown.” (Line 135-136).***

**Our modeling results suggest that paranoia does not result in an optimal estimate of the partner’s ability. In experiment 1, we find that high paranoia participants have high variance (omega-s) in their perception of the accuracy of their partner. This high variance is definitely suboptimal – it’s not that they believe that the partner is either more correct than them, or less correct than them – highly paranoid people infer that their partners change their minds more. Furthermore, we show in experiment 2 that this does not affect self-deception (Lines 367-402). In this experiment the partner’s bets are correct more of the time (75%, vs 50% in experiment 1), and we see the high paranoia individuals have a decrease in their variance estimation down to the levels of low paranoia participants. However, we still see higher self-deception (and higher reported confidence) in high paranoia individuals (Supplementary Figure 3). Therefore, we do not believe that the subject’s assessment of the partner’s accuracy contributes significantly to self-deception.**

It is possible that the experimental design accounts for this possibility, but if it does, it was not clear to me. Please clarify why the subjects should not respond against the partner in the competition condition and with the partner in the cooperation condition, when the stimulus is ambiguous (50%). Please also clarify why paranoia could not also be affecting the subject’s assessment of the partner’s accuracy.

**Please see above for our response to your comments. We feel as though this issue is addressed with experiment 2.**

Reviewer #3: Thanks to the authors for responding to my comments - I have only some minor remaining points that don't require re-review.

Regarding the concept of 'self-deception' in this experiment. I had not grasped that the partner is betting on an image *that they have not seen either* - can this be made as clear as possible in the methods? I had assumed the partner had seen it but the player had not.

**We have made this clearer in the methods (and have addressed it specifically in response to reviewer 2). We do not tell participants that the partner has been given any special or extra information than them and crucially the bets are made before the image is seen (we tell them that the bet is made before they see the image). Please see lines 135-136.**

The addition to the discussion: "Paranoia was found to be unrelated to betrayal aversion – when one has a higher aversion to risky situations where outcomes are contingent upon social factors compared to non-social factors which does support the coalitional cognition model". Is this correct or do the authors mean this does NOT support the coalitional model? I am not sure why absence of betrayal aversion supports that model?

**Thank you for catching this typo – we did indeed mean to claim that the data referenced do NOT support coalitional accounts.**

About the parameter recovery - I hope it does not become common practice to only report p-values: these will be strongly affected by the number of simulations conducted and so are a bit meaningless. The correlation is really the key measure

**We completely agree. Both p-values and correlations were reported in the text (lines 420-421).**
